# Perampanel as monotherapy or first adjunctive therapy in pediatric and adult patients with epilepsy: the first United States-based phase IV open-label ELEVATE study

**DOI:** 10.1007/s00415-024-12399-w

**Published:** 2024-05-10

**Authors:** Vineet Punia, Pavel Klein, Temenuzhka Mihaylova, Victor Biton, Omar Samad, Leock Y. Ngo, Dinesh Kumar, Manoj Malhotra

**Affiliations:** 1 Epilepsy Center, Cleveland Clinic, Cleveland, OH USA; 2 Mid-Atlantic Epilepsy and Sleep Center, Bethesda, MD USA; 3 University of Michigan, Ann Arbor, MI USA; 4 Arkansas Epilepsy Program, Little Rock, AR USA; 5 Eisai Inc., Nutley, NJ USA; 6 Formerly: Eisai Inc., Nutley, NJ USA

**Keywords:** Combination drug therapy, Focal-onset seizures, Generalized tonic–clonic seizures, Monotherapy, Perampanel, Refractory epilepsy

## Abstract

ELEVATE (Study 410; NCT03288129) is the first prospective, multicenter, open-label, Phase IV study of perampanel as monotherapy or first adjunctive therapy in patients aged ≥ 4 years with focal-onset seizures or generalized tonic–clonic seizures in the United States. The study included Screening, Titration (≤ 13 weeks), Maintenance (39 weeks), and Follow-up (4 weeks) Periods. During Titration, perampanel was initiated at 2 mg/day and up-titrated to 4 mg/day at Week 3. Depending on response and tolerability, optional up-titrations to a maximum of 12 mg/day occurred. The primary endpoint was retention rate; additional endpoints included seizure-freedom rate, 50% responder rate, and incidence of treatment-emergent adverse events (TEAEs). At baseline, 10 (18.5%) patients were assigned to the monotherapy group and 44 (81.5%) patients to the first adjunctive therapy group. However, due to the addition of an anti-seizure medication along with perampanel on the first day of treatment, one patient was excluded from the monotherapy subgroup analyses. The mean perampanel exposure duration was 39.8 weeks and 32 (59.3%) patients completed the study. Retention rate at 12 months (or study completion) was 63.0% (monotherapy, 77.8%; first adjunctive therapy, 59.1%). Seizure-freedom rate during the Maintenance Period was 32.7% (monotherapy, 44.4%; first adjunctive therapy, 29.5%) and the 50% responder rate was 78.7% (monotherapy, 85.7%; first adjunctive therapy, 76.9%). TEAEs and serious TEAEs were reported by 88.9% (n = 48/54) and 7.4% (n = 4/54) of patients, respectively. Overall, the efficacy and safety of perampanel as monotherapy or first adjunctive therapy support the use of perampanel as early-line treatment for epilepsy.

**Supplementary Information:**

The online version contains supplementary material available at 10.1007/s00415-024-12399-w.

## Introduction

Early response to anti-seizure medication (ASM) is associated with improved prognosis in patients with epilepsy [1]. The failure to control seizures with two or more ASMs (administered as monotherapy or adjunctive therapy) reduces the likelihood of achieving seizure control with subsequent ASMs [2, 3]; however, polytherapy can increase treatment-emergent adverse events (TEAEs), drug interactions between ASMs, psychiatric and behavioral side effects (such as depression), and noncompliance [4–6]. In addition, polytherapy may increase seizure frequency and therefore impact patient quality of life (QoL) [7]. There are limited data regarding the efficacy or effectiveness of ASMs administered as monotherapy in adults with focal-onset seizures (FOS) or generalized tonic–clonic seizures (GTCS) [8]. To improve the QoL and prognosis for patients with FOS and/or GTCS, further research is required to explore the efficacy of ASMs administered as monotherapy or early adjunctive therapy.

Perampanel, a selective, non-competitive α-amino-3-hydroxy-5-methyl-4-isoxazolepropionic acid (AMPA) receptor antagonist, is a once-daily oral ASM for FOS and GTCS [9, 10]. In the United States (US), perampanel is approved for the treatment of FOS (adjunctive and monotherapy), with or without focal to bilateral tonic–clonic seizures (FBTCS), in patients aged ≥ 4 years, and as adjunctive treatment of GTCS in patients aged ≥ 12 years [9]. The approval of perampanel as monotherapy was based on the US Food and Drug Administration’s regulatory pathway for monotherapy use, whereby efficacy and safety data were extrapolated from three Phase III studies of adjunctive perampanel in patients with treatment-resistant FOS, with or without FBTCS [11–13]. In a recent pooled analysis of data from 44 real-world studies of perampanel (PERMIT), it was found that fewer previous ASMs or concomitant ASMs at baseline were associated with improved seizure control and reduced likelihood of adverse events (AEs) [14]. In addition, emerging data from open-label and real-world studies investigating the efficacy and safety of perampanel as monotherapy [15–19] or first adjunctive treatment [19–21] support its use as an early-line treatment for patients who are treatment naïve or have less refractory epilepsy. However, further research regarding the clinical use of perampanel as monotherapy or first adjunctive therapy is required to improve the management of epilepsy in patients.

To address this, we report the efficacy and safety results from ELEVATE (Study 410; NCT03288129), the first prospective study of perampanel administered as monotherapy or first adjunctive therapy in a clinical setting in patients aged ≥ 4 years with FOS, with or without FBTCS, or GTCS in the US.

## Methods

### Study design

**Fig. 1:**
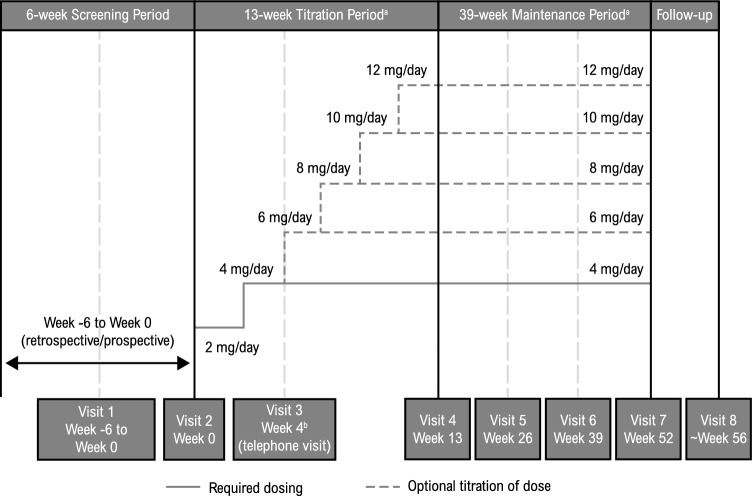
ELEVATE study design. **a** Treatment Phase: 52 weeks. **b** Patients were contacted via telephone by the investigator at Week 4, then bi-weekly as necessary. Dose adjustments and rationale, as well as adverse events, were recorded in the case report form

ELEVATE was a multicenter, open-label, Phase IV study of oral perampanel (tablets) as monotherapy or first adjunctive therapy in patients aged ≥ 4 years with FOS, with or without FBTCS, or with GTCS conducted at 14 sites in the US between August 23, 2017 and April 27, 2021. The study consisted of a Screening Period (up to 6 weeks prior to the first perampanel dose), Titration Period (up to 13 weeks), Maintenance Period (39 weeks), and Follow-up Period (4 weeks; Fig. 1).

During the Titration Period, perampanel was initiated at 2 mg/day and up-titrated to 4 mg/day at Week 3; additional up-titrations (to a maximum of 12 mg/day in 2-mg increments at intervals of ≥ 2 weeks) were optional based on clinical response and tolerability. Patients who concomitantly received enzyme-inducing ASMs (phenytoin, carbamazepine, oxcarbazepine, or eslicarbazepine) could be up-titrated in increments of 2 mg at 1-week intervals. During the Maintenance Period, patients continued to receive the perampanel dose that was administered at the end of the Titration Period; perampanel dose could be adjusted (to a maximum of 12 mg/day) during the Maintenance Period depending on clinical response and tolerability. Any patients unable to tolerate 4 mg/day by the end of the Titration Period or during the Maintenance Period were discontinued from the study.

### Patients

Patients were eligible for the study if they had experienced either two unprovoked (or reflex) seizures > 24 h apart or had experienced one unprovoked (or reflex) seizure with electroencephalogram evidence of seizures, and were either treatment naïve or required adjunctive therapy following failure to control seizures with ASM monotherapy. It was recommended that patients who received perampanel as first adjunctive therapy be on a stable dose of one concomitant ASM for ≥ 8 weeks prior to perampanel initiation and throughout the 13-week Titration Period. Dose and administration of the concomitant ASM could be modified during the Maintenance Period as per the investigator’s judgment. The main exclusion criteria were previous or current treatment with perampanel, presence or history of Lennox-Gastaut syndrome, and presence of non-motor focal aware seizures only.

### Efficacy assessments

The primary endpoint of the study was retention rate at 3, 6, 9, and 12 months (or study completion) in the Safety Analysis Set (SAS), defined as all patients who received at least one dose of perampanel and had at least one post-dose safety assessment. The retention rate refers to the number of patients who remained on perampanel (either as monotherapy or first adjunctive therapy) at the above specified time-points. The secondary endpoints included seizure freedom (proportion of patients who achieve seizure-free status for FOS, FBTCS, and GTCS during the Maintenance Period) in the Full Analysis Set (FAS), defined as patients who received at least one dose of perampanel and had at least one post-dose seizure measurement. Exploratory endpoints included 50% responder rate (patients who have ≥ 50% reduction in seizure frequency relative to baseline) during the Maintenance Period and median percent change in seizure frequency per 28 days during the Titration and Maintenance Periods relative to baseline; assessed in a subset of the FAS with sufficient baseline seizure frequency data. Seizure frequency was based on both prospective counts (seizure counts at baseline and thereafter in seizure diaries) and retrospective counts (seizure counts during the 12-week period prior to first perampanel dose). Last observation carried forward (LOCF) type imputation was employed to handle missing data for seizure-related efficacy endpoints. Up to 2 months of titration data were used for imputation for patients who dropped out early in the Maintenance Period.

### Safety and tolerability assessments

Secondary endpoints included the assessment of the incidence of TEAEs, treatment-related TEAEs, serious TEAEs, TEAEs leading to perampanel dose adjustment, and most common TEAEs. In addition, discontinuation from treatment, prior and concomitant ASM, cognition, suicidality or depressive symptoms, perampanel dosage and exposure, and compliance were monitored throughout the study. The incidence of suicidal behavior and suicidal ideation (i.e., suicidality) were monitored using the Columbia-Suicide Severity Rating Scale (C-SSRS) in patients aged ≥ 6 years. C-SSRS scores were evaluated by the investigator and an isolated suicidality rating scale response was classified as a TEAE per the investigator’s judgment. Patients under 6 years old were clinically monitored for suicidality.

### Exploratory endpoints

Additional exploratory endpoints investigated the proportion of patients with cognitive impairment relative to baseline. Cognition was assessed using the EpiTrack® screening tool (or EpiTrack® Junior in patients aged ≥ 6 to 16 years), scored by the investigator, to clinically monitor adverse cognitive effects associated with ASMs [22]. To aid the interpretation of the cognition score, depression was assessed using the Beck Depression Inventory-II (BDI-II) [23]. Additionally, change in subjective sleep quality was assessed using the retrospective sleep quality instrument, the Pittsburgh Sleep Quality Index (PSQI) [24], and QoL was assessed using the epilepsy-specific QoL in Epilepsy Inventory-31 [QOLIE-31] survey. The details of each assessment and scoring parameters are presented in Supplementary Table 1.

### Post hoc analysis of patients with a history of psychiatric and behavioral events

Psychiatric and behavioral side effects have been associated with the use of ASMs in patients with epilepsy, and evidence suggests that patients with a history of psychiatric events may be predisposed to these side effects [25–27]. Therefore, a post hoc analysis was performed to assess safety and the mean change from baseline in cognition (EpiTrack®) scores at Week 52 in a subgroup of patients with a history of psychiatric and behavioral events (as defined by Medical Dictionary for Regulatory Activities [MedDRA]) from ELEVATE.

### Statistical analysis

As this study did not have a control arm, only descriptive statistics were performed. Efficacy and safety outcomes are summarized by treatment group (overall population, monotherapy group [patients who either received no ASMs at baseline or received other ASMs and converted to perampanel only at baseline], and first adjunctive therapy group) and by seizure type (FOS [patients with FOS only, with or without FBTCS] and GTCS [patients with GTCS only]).

## Results

### Patients

The study enrolled 68 patients; two (2.9%) patients were aged 4 to < 12 years, seven (10.3%) were aged 12 to < 18 years, 53 (77.9%) were aged 18 to 64 years, and six (8.8%) were aged > 64 years. Of these, 54 patients aged ≥ 12 years were treated with perampanel and included in the SAS, and 52 patients were included in the FAS. Patient demographics and clinical characteristics during baseline are presented in Table 1. The mean (standard deviation [SD]) age of patients in the overall population was 38.5 (17.3) years; four (7.4%) patients were aged 12 to < 18 years, 44 (81.5%) patients were aged 18 to 64 years, and six (11.1%) patients were aged > 64 years. The mean (SD) time since epilepsy diagnosis in the overall population was 6.2 (9.1) years.

**Table 1:** Patient demographics and clinical characteristics during baseline by treatment group and seizure type (Safety Analysis Set)

	Perampanel monotherapy^a^			Perampanel first adjunctive therapy^b^			Overall^c^		
	FOS^d^ (*n* = 7)	GTCS^d^ (*n* = 2)	Total^d^ (*N* = 9)	FOS^d^ (*n* = 30)	GTCS^d^ (*n* = 9)	Total^d^ (*N* = 44)	FOS^d^ (*n* = 38)	GTCS^d^ (*n* = 11)	Total^d^ (*N* = 54)
Mean (SD) age,^e^ years	37.6 (20.2)	28.0 (1.4)	35.4 (18.0)	42.6 (18.2)	25.1 (10.3)	38.8 (17.4)	42.0 (18.3)	25.6 (9.3)	38.5 (17.3)
Age group, *n* (%)									
12 to < 18 years	0 (0.0)	0 (0.0)	0 (0.0)	1 (3.3)	3 (33.3)	4 (9.1)	1 (2.6)	3 (27.3)	4 (7.4)
18 to 64 years	6 (85.7)	2 (100.0)	8 (88.9)	24 (80.0)	6 (66.7)	35 (79.5)	31 (81.6)	8 (72.7)	44 (81.5)
> 64 years	1 (14.3)	0 (0.0)	1 (11.1)	5 (16.7)	0 (0.0)	5 (11.4)	6 (15.8)	0 (0.0)	6 (11.1)
Female, *n* (%)	2 (28.6)	1 (50.0)	3 (33.3)	18 (60.0)	2 (22.2)	23 (52.3)	21 (55.3)	3 (27.3)	27 (50.0)
Race, *n* (%)									
Asian	0 (0.0)	0 (0.0)	0 (0.0)	0 (0.0)	1 (11.1)	1 (2.3)	0 (0.0)	1 (9.1)	1 (1.9)
Black or African American	2 (28.6)	0 (0.0)	2 (22.2)	4 (13.3)	2 (22.2)	6 (13.6)	7 (18.4)	2 (18.2)	9 (16.7)
White	5 (71.4)	2 (100.0)	7 (77.8)	24 (80.0)	6 (66.7)	35 (79.5)	29 (76.3)	8 (72.7)	42 (77.8)
Other	0 (0.0)	0 (0.0)	0 (0.0)	2 (6.7)	0 (0.0)	2 (4.5)	2 (5.3)	0 (0.0)	2 (3.7)
Mean (SD) time since epilepsy diagnosis,^f^ years	0.5 (1.0)	7.9 (11.1)	2.2 (5.2)	6.6 (10.5)	9.1 (8.1)	7.1 (9.6)	5.3 (9.6)	8.9 (8.1)	6.2 (9.1)
Etiology, *n* (%)									
Head injury/cranial trauma	1 (14.3)	0 (0.0)	1 (11.1)	3 (10.0)	0 (0.0)	3 (6.8)	4 (10.5)	0 (0.0)	4 (7.4)
Structural brain anomalies or malformations	0 (0.0)	0 (0.0)	0 (0.0)	1 (3.3)	0 (0.0)	1 (2.3)	1 (2.6)	0 (0.0)	1 (1.9)
Genetics	0 (0.0)	1 (50.0)	1 (11.1)	0 (0.0)	4 (44.4)	4 (9.1)	0 (0.0)	5 (45.5)	5 (9.3)
Family history	0 (0.0)	0 (0.0)	0 (0.0)	1 (3.3)	0 (0.0)	1 (2.3)	1 (2.6)	0 (0.0)	1 (1.9)
Other	0 (0.0)	0 (0.0)	0 (0.0)	2 (6.7)	1 (11.1)	3 (6.8)	2 (5.3)	1 (9.1)	3 (5.6)
Unknown	6 (85.7)	1 (50.0)	7 (77.8)	23 (76.7)	4 (44.4)	32 (72.7)	30 (78.9)	5 (45.5)	40 (74.1)

^a^Patients who either received no ASMs at baseline or received another ASM and converted to perampanel only at baseline. One patient was excluded from the monotherapy subgroup analyses due to the addition of an ASM along with perampanel on the first day of treatment

^b^Patients who received perampanel as first adjunctive therapy at baseline

^c^Patients who either received perampanel monotherapy or perampanel as first adjunctive therapy at baseline

^d^FOS includes patients with FOS only (with or without FBTCS); GTCS includes patients with GTCS only. The “Total” column includes patients with FOS only, GTCS only, and mixed FOS and GTCS; therefore, the total number of patients is greater than the sum of patients with FOS only or GTCS only

^e^Age at date of consent/assent

^f^Time from diagnosis to date of consent/assent

*ASM* anti-seizure medication, *FBTCS* focal to bilateral tonic–clonic seizures, *FOS* focal-onset seizures, *GTCS* generalized tonic–clonic seizures, *NA* not applicable, *SD* standard deviation

**Fig. 2:**
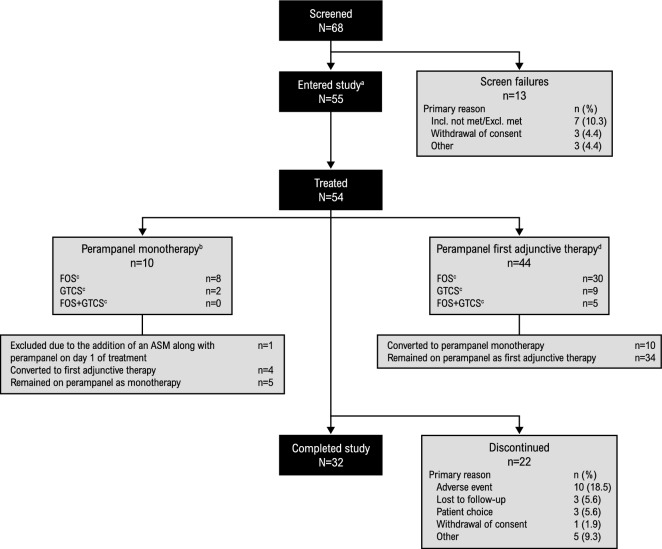
Patient disposition. **a** One patient did not take perampanel. **b** Patients who either received no ASMs at baseline or received another ASM and converted to perampanel only at baseline. One patient was excluded from the monotherapy subgroup analyses due to the addition of an ASM along with perampanel on the first day of treatment. **c** FOS includes patients with FOS only (with or without FBTCS); GTCS includes patients with GTCS only; FOS + GTCS includes patients with mixed FOS and GTCS. **d** Patients who received perampanel as first adjunctive therapy at baseline. *ASM* anti-seizure medication, *Excl* exclusion criteria, *FBTCS* focal to bilateral tonic–clonic seizures, *FOS* focal-onset seizures, *GTCS* generalized tonic–clonic seizures, *Incl* inclusion criteria

Out of the 54 patients in the SAS, 10 (18.5%) patients were assigned to the perampanel monotherapy group (Fig. 2). However, due to the addition of an ASM along with perampanel on the first day of treatment, one patient was excluded from the monotherapy subgroup analyses; the other nine patients in the monotherapy group were treatment naïve prior to perampanel initiation. The remaining 44 (81.5%) patients in the SAS received perampanel as first adjunctive therapy at baseline (Fig. 2); the most common concomitant ASMs at baseline were levetiracetam (54.5% [n = 24/44]) and lamotrigine (13.6% [n = 6/44]). During the study, four (44.4%) patients in the monotherapy group added another ASM and five (55.6%) patients remained on perampanel monotherapy for the entire study; 10 (22.7%) patients in the first adjunctive therapy group converted to monotherapy and 34 (77.3%) remained on perampanel as first adjunctive therapy. In total, 32 (59.3%) patients completed the study; the most common reason for discontinuation was AEs (18.5% [n = 10/54]) (Fig. 2). No patient who completed the study reported developing drug-resistant epilepsy.

The mean (SD) duration of exposure to perampanel was 39.8 (20.0) weeks in the overall population. In the perampanel monotherapy and first adjunctive therapy groups, the mean (SD) duration of exposure to perampanel was 45.1 (17.0) weeks and 38.3 (20.6) weeks, respectively. The median (minimum, maximum) daily dose of perampanel was 4.0 (4, 12) mg in the monotherapy group and 6.0 (4, 11) mg in the first adjunctive therapy group during the Maintenance Period.

### Efficacy outcomes

**Fig. 3:**
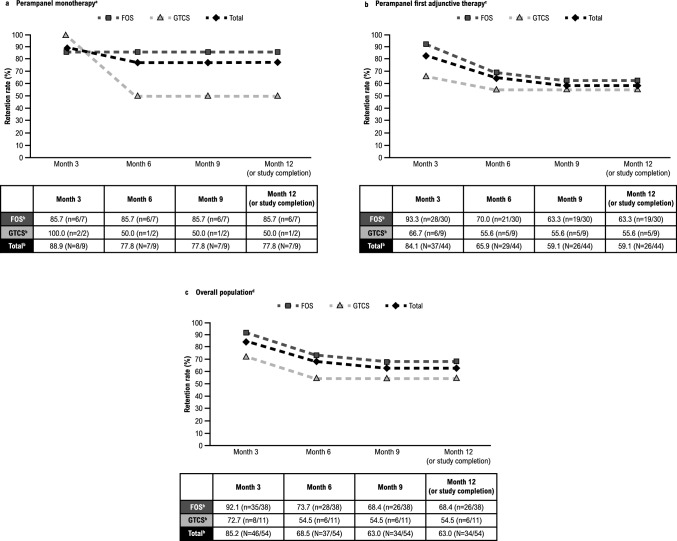
Retention rate of perampanel at 3, 6, 9, and 12 months (or study completion) by treatment group ([**a**] perampanel monotherapy, [**b**] perampanel first adjunctive therapy, and [**c**] overall population) and seizure type (Safety Analysis Set). **a** Patients who either received no ASMs at baseline or received another ASM and converted to perampanel only at baseline. One patient was excluded from the monotherapy subgroup analyses due to the addition of an ASM along with perampanel on the first day of treatment. **b** FOS includes patients with FOS only (with or without FBTCS); GTCS includes patients with GTCS only. The “Total” column includes patients with FOS only, GTCS only, and mixed FOS and GTCS; therefore, the total number of patients is greater than the sum of patients with FOS only or GTCS only. **c** Patients who received perampanel as first adjunctive therapy at baseline. **d** Patients who either received perampanel monotherapy or perampanel as first adjunctive therapy at baseline. *ASM* anti-seizure medication, *FBTCS* focal to bilateral tonic–clonic seizures, *FOS* focal-onset seizures, *GTCS* generalized tonic–clonic seizures

**Fig. 4:**
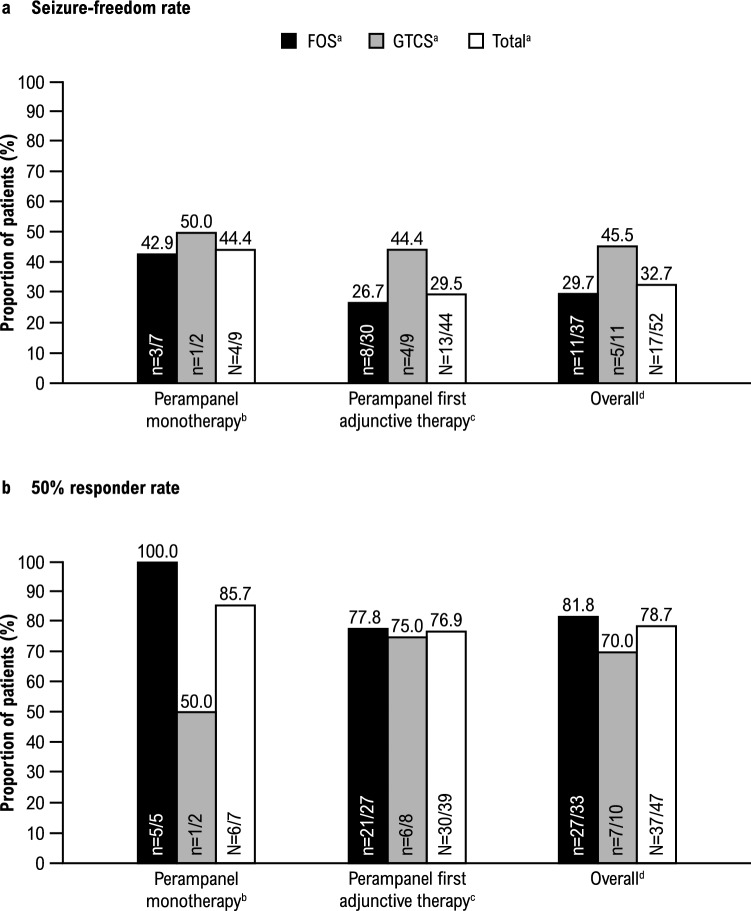
**a** Seizure-freedom rate and **b** 50% responder rate during the entire Maintenance Period (LOCF) by treatment group and seizure type (Full Analysis Set). Percentages are based on the total number of patients with non-missing values. **a** FOS includes patients with FOS only (with or without FBTCS); GTCS includes patients with GTCS only. The “Total” column includes patients with FOS only, GTCS only, and mixed FOS and GTCS; therefore, the total number of patients is greater than the sum of patients with FOS only or GTCS only. **b** Patients who either received no ASMs at baseline or received another ASM and converted to perampanel only at baseline. One patient was excluded from the monotherapy subgroup analyses due to the addition of an ASM along with perampanel on the first day of treatment. **c** Patients who received perampanel as first adjunctive therapy at baseline. **d** Patients who either received perampanel monotherapy or perampanel as first adjunctive therapy at baseline. *ASM* anti-seizure medication, *FBTCS* focal to bilateral tonic–clonic seizures, *FOS* focal-onset seizures, *GTCS* generalized tonic–clonic seizures, *LOCF* last observation carried forward

Retention rates at 3, 6, 9, and 12 months (or study completion) are presented by treatment group and seizure type in Fig. 3. Retention rate in the overall population at 12 months (or study completion) was 63.0% (n = 34/54; monotherapy group, 77.8% [n = 7/9]; first adjunctive therapy group, 59.1% [n = 26/44]). The seizure-freedom rate and 50% responder rate are presented in Fig. 4 by treatment group and seizure type; seizure freedom was achieved by 32.7% (n = 17/52) of patients and 50% response was achieved by 78.7% (n = 37/47) of patients in the overall population during the Maintenance Period. Seizure freedom and 50% response was achieved by 44.4% (n = 4/9) and 85.7% (n = 6/7) of patients in the monotherapy group, respectively; similarly, 29.5% (n = 13/44) of patients in the first adjunctive therapy group achieved seizure freedom and 76.9% (n = 30/39) of patients achieved 50% response. The median (minimum, maximum) reduction in total-seizure frequency per 28 days during the Titration and Maintenance Periods in the overall population was 86.8% (-2700.0, 100.0) and 77.9% (-2700.0, 100.0), respectively (Supplementary Fig. 1).

### Safety outcomes

An overview of TEAEs and most common TEAEs by treatment group and seizure type is presented in Table 2. TEAEs were reported by 88.9% (n = 48/54) of patients in the overall population (monotherapy group, 77.8% [n = 7/9]; first adjunctive therapy group, 90.9% [n = 40/44]). The incidence of TEAEs was lower during the Maintenance Period compared with the Titration Period across seizure types (Supplementary Table 2); in total, 87.0% (n = 47/54) of patients reported TEAEs during the Titration Period and 54.5% (n = 24/44) of patients reported TEAEs during the Maintenance Period.

**Table 2:** Overview of TEAEs and most common TEAEs (occurring in ≥ 10% of patients in the overall population) by treatment group and seizure type (Safety Analysis Set)

	Perampanel monotherapy^a^			Perampanel first adjunctive therapy^b^			Overall^c^		
	FOS^d^ (*n* = 7)	GTCS^d^ (*n* = 2)	Total^d^ (*N* = 9)	FOS^d^ (*n* = 30)	GTCS^d^ (*n* = 9)	Total^d^ (*N* = 44)	FOS^d^ (*n* = 38)	GTCS^d^ (*n* = 11)	Total^d^ (*N* = 54)
TEAEs, *n* (%)	5 (71.4)	2 (100.0)	7 (77.8)	26 (86.7)	9 (100.0)	40 (90.9)	32 (84.2)	11 (100.0)	48 (88.9)
Treatment-related TEAEs, *n* (%)	4 (57.1)	2 (100.0)	6 (66.7)	17 (56.7)	6 (66.7)	27 (61.4)	22 (57.9)	8 (72.7)	34 (63.0)
Serious TEAEs, *n* (%)	0 (0.0)	1 (50.0)	1 (11.1)	2 (6.7)	1 (11.1)	3 (6.8)	2 (5.3)	2 (18.2)	4 (7.4)
TEAEs leading to perampanel dose adjustment, *n* (%)									
Discontinuation	1 (14.3)	1 (50.0)	2 (22.2)	6 (20.0)	2 (22.2)	9 (20.5)	6 (15.8)	3 (27.3)	10 (18.5)
Dose reduction	1 (14.3)	0 (0.0)	1 (11.1)	1 (3.3)	0 (0.0)	2 (4.5)	2 (5.3)	0 (0.0)	3 (5.6)
Most common TEAEs,^e^ *n* (%)									
Dizziness	1 (14.3)	0 (0.0)	1 (11.1)	9 (30.0)	5 (55.6)	14 (31.8)	10 (26.3)	5 (45.5)	15 (27.8)
Fatigue	2 (28.6)	0 (0.0)	2 (22.2)	3 (10.0)	3 (33.3)	6 (13.6)	6 (15.8)	3 (27.3)	9 (16.7)
Somnolence	1 (14.3)	0 (0.0)	1 (11.1)	4 (13.3)	1 (11.1)	6 (13.6)	6 (15.8)	1 (9.1)	8 (14.8)
Vomiting	0 (0.0)	1 (50.0)	1 (11.1)	2 (6.7)	2 (22.2)	5 (11.4)	2 (5.3)	3 (27.3)	6 (11.1)

A patient with ≥ 2 TEAEs in the same category is only counted once

^a^Patients who either received no ASMs at baseline or received another ASM and converted to perampanel only at baseline. One patient was excluded from the monotherapy subgroup analyses due to the addition of an ASM along with perampanel on the first day of treatment

^b^Patients who received perampanel as first adjunctive therapy at baseline

^c^Patients who either received perampanel monotherapy or perampanel as first adjunctive therapy at baseline

^d^FOS includes patients with FOS only (with or without FBTCS); GTCS includes patients with GTCS only. The “Total” column includes patients with FOS only, GTCS only, and mixed FOS and GTCS; therefore, the total number of patients is greater than the sum of patients with FOS only or GTCS only

^e^TEAEs that occurred in ≥ 10% of patients in the overall population

*ASM* anti-seizure medication, *FBTCS* focal to bilateral tonic–clonic seizures, *FOS* focal-onset seizures, *GTCS* generalized tonic–clonic seizures, *TEAE* treatment-emergent adverse event

TEAEs leading to perampanel dose adjustment occurred in 13 (24.1%) patients in the overall population (monotherapy group, 22.2% [n = 2/9]; first adjunctive therapy group, 25.0% [n = 11/44]). The most common TEAEs were dizziness (overall population, 27.8% [n = 15/54]; monotherapy group, 11.1% [n = 1/9]; first adjunctive therapy group, 31.8% [n = 14/44]) and fatigue (overall population, 16.7% [n = 9/54]; monotherapy group, 22.2% [n = 2/9]; first adjunctive therapy group, 13.6% [n = 6/44]). The incidence of TEAEs leading to perampanel dose adjustment in patients taking concomitant levetiracetam or lamotrigine (the most common concomitant ASMs in the first adjunctive group) was 29.0% (n = 9/31).

Serious TEAEs were reported in four (7.4%) patients (Table 2); these were sudden unexpected death in epilepsy (SUDEP; n = 1 [FOS]), worsening depression and suicidal ideation (n = 1 [GTCS]), transient ischemic attack (n = 1 [FOS]), and mental status changes (n = 1, [GTCS]). The event of SUDEP was reported in a patient receiving perampanel as first adjunctive therapy with concomitant oxcarbazepine and was not related to perampanel. The events of worsening depression and suicidal ideation were reported in a patient receiving perampanel monotherapy (after transitioning from other ASMs to perampanel monotherapy at baseline); these events led to discontinuation and were considered to be related to perampanel. This patient had a history of ongoing major depression disorder and was receiving concomitant anti-depressant medication.

In the overall population, 33.3% (n = 18/54) of patients reported psychiatric AEs. Three (5.6%) patients (FOS, n = 2 [one with FBTCS]; GTCS, n = 1) experienced on-treatment suicidal ideation, as indicated by ≥ 1 positive response for suicidal ideation on the C-SSRS. Two of these patients had a history of suicidal ideation at baseline; both patients recovered without dose adjustment, and their suicidal ideation TEAEs resolved. The third patient (mentioned in the above paragraph) discontinued perampanel treatment; following discontinuation, the patient recovered, and the suicidal ideation resolved. No patients experienced on-treatment suicidal behavior.

### Exploratory endpoints

Additional exploratory endpoints are presented by seizure type in Table 3. In the overall population, there was a negative change in total EpiTrack® (n = 28), BDI-II (n = 29), and PSQI (n = 30) scores at 12 months compared to baseline (mean [SD] change from baseline in total score: EpiTrack® -0.4 [3.3], BDI-II -1.2 [7.9], PSQI -0.2 [3.9]) and an improvement in QOLIE (n = 29) total score at 12 months compared to baseline (mean [SD] change from baseline in total score, 2.4 [9.5]). However, these changes in total score were not clinically significant (Supplementary Table 1). The PSQI and QOLIE-31 individual domain results are presented in Supplementary Table 3.

**Table 3:** Overview of EpiTrack®, BDI-II, PSQI, and QOLIE-31 scores by seizure type (Safety Analysis Set)

	FOS^a^ (*n* = 38)	GTCS^a^ (*n* = 11)	Total^a^ (*N* = 54)
EpiTrack® total score,^b^ mean (SD)			
Baseline	33.9 (6.3); *n* = 37	32.1 (3.9); *n* = 10	33.4 (5.9); *n* = 52
Month 12	33.2 (5.3); *n* = 22	33.8 (4.1); *n* = 5	33.3 (4.9); *n* = 28
Change from baseline	− 0.6 (3.3); *n* = 22	1.6 (1.7); *n* = 5	− 0.4 (3.3); *n* = 28
BDI-II total score, mean (SD)			
Baseline	8.0 (8.5); *n* = 37	10.0 (11.5); *n* = 8	9.0 (9.0); *n* = 50
Month 12	6.7 (7.8); *n* = 24	5.0 (7.4); *n* = 5	6.6 (7.6); *n* = 30
Change from baseline	− 1.8 (8.0); *n* = 24	2.8 (8.3); *n* = 4	− 1.2 (7.9); *n* = 29
PSQI total score, mean (SD)			
Baseline	6.5 (4.1); *n* = 37	7.4 (2.7); *n* = 11	6.8 (3.7); *n* = 53
Month 12	6.0 (3.1); *n* = 24	6.2 (5.5); *n* = 6	6.2 (3.6); *n* = 31
Change from baseline	− 0.3 (3.9); *n* = 23	− 0.2 (4.2); *n* = 6	− 0.2 (3.9); *n* = 30
QOLIE-31 total score, mean (SD)			
Baseline	67.9 (15.3); *n* = 37	59.4 (23.7); *n* = 8	64.7 (18.0); *n* = 50
Month 12	71.3 (15.1); *n* = 24	74.4 (21.9); *n* = 4	70.7 (16.5); *n* = 29
Change from baseline	1.7 (10.0); *n* = 24	7.0 (6.4); *n* = 4	2.4 (9.5); *n* = 29

Only patients with non-missing data at both baseline and the relevant post-baseline visit are included in the change from baseline summary statistics

^a^FOS includes patients with FOS only (with or without FBTCS); GTCS includes patients with GTCS only. The “Total” column includes patients with FOS only, GTCS only, and mixed FOS and GTCS; therefore, the total number of patients is greater than the sum of patients with FOS only or GTCS only

^b^Age-corrected total score derivation: 16–20 years =  + 1 point; 21–35 years = no correction; 36–45 years =  + 1 point; 46–50 years =  + 3 points; 51–65 years =  + 4 points; 66–70 years =  + 6 points; > 70 years =  + 7 points

*BDI-II* Beck Depression Inventory-II, *FBTCS* focal to bilateral tonic–clonic seizures, *FOS* focal-onset seizures, *GTCS* generalized tonic–clonic seizures, *PSQI* Pittsburgh Sleep Quality Index, *QOLIE-31* Quality of Life in Epilepsy Inventory-31, *SD* standard deviation

### Post hoc analysis of patients with a history of psychiatric and behavioral events

There were 24 patients with a history of psychiatric or behavioral events, based on clinical history, included in the post hoc analysis. Five (20.8%) patients received perampanel monotherapy and 19 (79.2%) patients received perampanel as first adjunctive therapy. The most common concomitant ASM during the study was levetiracetam (37.5% [n = 9/24]). The results of the post hoc analysis are presented in Supplementary Table 4. The incidence of any TEAE in this subgroup was 95.8% (n = 23/24); 41.7% (n = 10/24) reported psychiatric TEAEs, in comparison to 33.3% (n = 18/54) in the overall population. Four (44.4%) patients receiving concomitant levetiracetam reported a psychiatric TEAE. With respect to cognition, the mean (SD) change in EpiTrack® total score from baseline at 12 months was − 1.1 (3.1) in patients with a history of psychiatric or behavioral events; this was consistent with the mean change in the overall population (− 0.4 [3.3]).

## Discussion

In this analysis of data from ELEVATE, perampanel monotherapy or first adjunctive therapy was associated with retention rates of 63.0%, median reductions in seizure frequency of 77.9%, and was generally well tolerated. Efficacy and safety data were similar between seizure types. Cognitive function, sleep quality, and QoL were not negatively impacted following perampanel monotherapy or first adjunctive therapy. Only one patient had de novo suicidal ideation which was reported as a TEAE, and no one experienced suicidal behavior. These findings are consistent with the known safety profile of perampanel. No new safety signals were observed [9]. Additionally, more than one in five patients (22.7%) in the first adjunctive therapy group were able to convert to perampanel monotherapy, thereby reducing medication burden.

Results observed in ELEVATE are in line with clinical evidence from other open-label and real-world studies assessing the efficacy and safety of perampanel in patients with epilepsy administered as monotherapy [15–19] or first adjunctive therapy [20, 21]. In ELEVATE, the retention rate at 12 months (or study completion) was 77.8% among patients receiving perampanel monotherapy, which is comparable to retention rates of 50.0–71.4% at 12 months (or study completion) in other real-world studies investigating efficacy of perampanel monotherapy [16–19]. Patients receiving perampanel as first adjunctive therapy in ELEVATE had a 50% responder rate of 76.9%, which is consistent with the 50% responder rate (80.0%) reported in the Phase IV Study 412 that evaluated the efficacy and safety of perampanel as a first adjunctive therapy in patients with FOS, with or without FBTCS [20].

In ELEVATE, 44.4% of patients receiving perampanel monotherapy achieved seizure-free status during the Maintenance Period, which is lower than that observed in FREEDOM (Study 342; NCT03201900), an open-label study that evaluated perampanel monotherapy among patients with newly diagnosed FOS, with or without FBTCS, or in patients who had relapsed [15]. In FREEDOM, seizure-freedom rates were 63.0% (4 mg/day) and 74.0% (4 or 8 mg/day) during the Maintenance Period. The higher seizure-freedom rate in the FREEDOM study could have been due to the shorter duration of the Maintenance Period (FREEDOM, 26 weeks; ELEVATE, 39 weeks) and the greater number of patients receiving perampanel monotherapy (FREEDOM, n = 89; ELEVATE, n = 9). Furthermore, the FREEDOM study only assessed patients with FOS, with or without FBTCS and included a greater proportion of patients who were treatment naïve or newly diagnosed which could contribute to the higher seizure-freedom rate in this study.

The safety profile reported for perampanel administered as monotherapy or first adjunctive therapy in ELEVATE was comparable to that described in other studies that evaluated perampanel either as monotherapy or adjunctive therapy including the FREEDOM study [15], Phase III studies of adjunctive perampanel [11–13], and the PERMIT study; a large, pooled analysis of real-world data [14]. Moreover, the incidence of serious TEAEs was low in patients receiving perampanel monotherapy (11.1%) or first adjunctive therapy (6.8%) in ELEVATE, which was again consistent with the findings of previous studies of perampanel monotherapy (0.0–10.1%) [15–19] or first adjunctive therapy (0.0–7.8%) [20, 21].

The safety profile of early-line perampanel reported here is comparable to that reported for other ASMs, such as lacosamide and cenobamate [28, 29]. Despite differences in retention rate (63% with perampanel in the overall population at 12 months (or study completion) and 79% with cenobamate at 12 months), the incidence of serious TEAEs was low with early-line perampanel (7.4%) and similar to that reported with adjunctive lacosamide (6.6%) and adjunctive cenobamate (8.1%). The differences in study design may explain the differences in retention rate between perampanel and cenobamate. In ELEVATE, patients who could not tolerate 4 mg/day perampanel by the end of the Titration Period or during the Maintenance Period were required, per study protocol, to withdraw from the study, likely accounting for the 10 patients who discontinued treatment due to AEs. In addition, the study design only allowed for one concomitant ASM, along with conversion to perampanel monotherapy or dose adjustment only, which could have resulted in discontinuations due to a lack of efficacy. Whereas in the phase 3 trial assessing the efficacy of of long-term adjunctive cenobamate, a high proportion of patients were receiving ≥ 2 concomitant ASMs (n = 1098, 82.0%) and, could remove, add, or adjust the dose of concomitant ASM as clinically required except for those receiving concomitant phenytoin or phenobarbital [29].

Cognitive function and QoL in patients with epilepsy can be negatively affected by poor sleep quality. The use of some ASMs can lead to disruptions in sleep quality, such as daytime sleepiness, which can worsen seizure control [30]. Here, we demonstrated that perampanel did not adversely affect sleep quality. In addition, cognitive function and QoL were not negatively impacted following perampanel monotherapy or first adjunctive therapy. These findings are supported by previous evidence that suggests perampanel can have a positive effect on sleep quality [30, 31].

In the overall ELEVATE population, 33.3% (n = 18/54) of patients reported psychiatric TEAEs. This result is in line with previous real-world studies of perampanel in which 15.4–28.5% of patients reported psychiatric TEAEs [14, 18, 19]. Among the 24 patients who had a history of psychiatric or behavioral events in ELEVATE, the incidence of psychiatric TEAEs was 41.7% (n = 10/24), which was numerically greater compared with the overall study population. These observations are in line with the evidence that patients with a history of psychiatric and behavioral events may be predisposed to psychiatric AEs [25, 26]. Moreover, psychiatric TEAEs are frequently reported with the use of perampanel, levetiracetam, and topiramate [32, 33]. In ELEVATE, 37.5% (n = 9/24) of patients with pre-existing psychiatric or behavioral conditions were receiving concomitant levetiracetam, of whom four (44.4%) reported psychiatric TEAEs. However, results from a post hoc analysis of pooled data (N = 1038) from four phase III trials assessing the effects of perampanel in patients already receiving 1–3 concomitant ASMs demonstrated that concomitant treatment with levetiracetam had no significant effect on the occurrence of psychiatric AEs in this patient population [34]. Interpretation of these data from ELEVATE may be limited due to the small number of patients with a history of psychiatric or behavioral events included in this post hoc analysis. Overall, this post hoc analysis showed that perampanel was well tolerated in patients with a history of psychiatric and behavioral events, however, all patients should be monitored for signs of psychiatric AEs and perampanel dose reductions may be considered to manage symptoms [26].

There are some limitations of ELEVATE that should be considered when interpretating the results. Firstly, this was an open-label study of perampanel as monotherapy or first adjunctive therapy without a placebo-control arm. A placebo group is deemed unethical for studies of ASM monotherapy because patients in this group would not receive any ASMs to regulate seizures which could be critical for epilepsy management [35]. In addition, the study faced recruitment challenges due to the COVID-19 pandemic, which severely limited the recruitment for more than a year. At this point, it was decided to stop enrollment and analyze the data; thus, the number of patients enrolled in ELEVATE was smaller than intended. Furthermore, the study was not designed with enrollment stratification to ensure a certain number of patients in each patient cohort, thus patient numbers in the subgroups were small, particularly in the monotherapy treatment group and across seizure types.

## Conclusion

ELEVATE is the first prospective study of perampanel administered as monotherapy or first adjunctive therapy in patients aged ≥ 4 years with FOS, with or without FBTCS, or GTCS in the US. Perampanel as monotherapy and as first adjunctive therapy was generally well tolerated and outcomes were consistent with the known safety profile of perampanel, with no new safety signals observed [9]. The data observed in the ELEVATE study contribute to emerging data that perampanel can be introduced as an early line treatment in patients with FOS or GTCS, rather than administrating after a patient has failed several ASMs.

### Supplementary Information

Below is the link to the electronic supplementary material.

Supplementary file1 (PDF 264 KB)

## Data Availability

The data that support the findings of this study are available from the corresponding author upon reasonable request.
