# Supplementary material for: Perampanel as monotherapy or first adjunctive therapy in pediatric and adult patients with epilepsy: the first United States-based phase IV open-label ELEVATE study
Source: J Neurol. 2024 May 10;271(7):4587–98. doi: 10.1007/s00415-024-12399-w (PMC11233369; doi:10.1007/s00415-024-12399-w)
Supplement: Supplementary file 1 — Supplementary file1 (PDF 264 KB) [file 415_2024_12399_MOESM1_ESM.pdf]

Perampanel as monotherapy or first adjunctive therapy in patients aged ≥4 years with epilepsy: the first United States-based phase IV open-label ELEVATE study

Vineet Punia,[1] Pavel Klein,[2] Temenuzhka Mihaylova,[3] Victor Biton,[4] Omar Samad,[5] Leock Y Ngo,[6] Dinesh Kumar,[5] Manoj Malhotra[6]

[1]Epilepsy Center, Cleveland Clinic, OH, USA; [2]Mid-Atlantic Epilepsy and Sleep Center, Bethesda, MD, USA; [3]University of Michigan, Ann Arbor, MI, USA; [4]Arkansas Epilepsy Program, Little Rock, AR, USA; [5]Eisai Inc., Nutley, NJ, USA; [6]Formerly: Eisai Inc., Nutley, NJ, USA

Corresponding author and contact details: Vineet Punia, Epilepsy Center, Cleveland Clinic, Ohio, USA.

E-mail address: Puniav@ccf.org

Supplementary Information

Supplementary Results

Efficacy outcomes

In the monotherapy group, median (minimum, maximum) total-seizure frequency per 28 days from baseline was reduced during the Titration (100.0% [-543.1, 100.0]) and Maintenance Periods (88.6% [-727.6, 100.0]). The patient with a median increase in seizure frequency of 543.1% and 727.6% during the Titration and Maintenance Periods, respectively, was one of two patients with GTCS and was likely due to seizure clusters; however, the second patient with GTCS in the monotherapy group showed a 100.0% median reduction in seizure frequency during both periods. In the first adjunctive therapy group, the median reduction (minimum, maximum) in total seizure frequency per 28 days was 87.3% (-2700.0, 100.0) during the Titration Period and 81.3% (-2700.0, 100.0) during the Maintenance Period.

Supplementary Table 1. Overview of EpiTrack®, BDI-II, PSQI, and QOLIE-31 assessments and scoring parameters

Exploratory endpoints	Age of patients assessed	Time of assessment	Details of each assessment	Scoring parameters	Clinically significant cut-offs
**EpiTrack®**	≥16 years<br>≥6–16 years (EpiTrack Junior)	Baseline, 3, and 12 months	Consists of six subtests: response inhibition, visuo-motor speed, mental flexibility, visual motor planning, verbal fluency, and working memory	Age-corrected[a] score calculated with a maximum of 49 points (≤28 points = significantly impaired; 29–31 = mildly impaired; 32–38 points = normal; ≥39 points = excellent)	Increase of three points or decrease of two points from baseline [22]
**BDI-II**	≥16 years	Baseline, 3, and 12 months	Consists of a 21-question, multiple choice, self-reported inventory	Each question answered using a four-point (0–3) scale, total score ranging between 0–63; higher scores indicate more severe depression	Increase of ≥3 points or decrease of two points from baseline [23]
**PSQI**	≥12 years	Baseline and 12 months	Consists of 19 items that are grouped into seven components: overall sleep quality, sleep latency, sleep duration, habitual sleep efficiency, sleep disturbances, use of sleeping medications, and daytime dysfunction	Global score (0–21) yielded by summing seven component scores (weighted equally on a scale of 0–3); higher scores indicate worse sleep quality	Score of >5 indicates severe difficulties in ≥2 areas or moderate difficulties in >3 areas [24]
**QOLIE-31**	≥18 years	Baseline and 12 months	Consists of 31 items assessing seven subdomains: overall QoL, seizure worry, emotional well-being, energy/fatigue, cognitive functioning, medication effects, and social functioning	Raw numeric values of items converted to scores of 0–100; higher scores reflect a better QoL [34]	5-point change in total score from baseline [35]

[a]Age-corrected total score derivation: 16–20 years = +1 point; 21–35 years = no correction; 36–45 years = +1 point; 46–50 years = +3 points; 51–65 years = +4 points; 66–70 years = +6 points; >70 years = +7 points

BDI-II Beck Depression Inventory-II, *PSQI* Pittsburgh Sleep Quality Index, *QOL* Quality of Life, *QOLIE-31* Quality of Life in Epilepsy Inventory-31

Supplementary Table 2. Overview of TEAEs and most common TEAEs (occurring in ≥10% of patients in total population in either treatment period) by seizure type and treatment period (Safety Analysis Set)

	**Titration Period[a]**			**Maintenance Period[b]**		
	**FOS[c] (n=38)**	**GTCS[c] (n=11)**	**Total[c] (N=54)**	**FOS[c] (n=33)**	**GTCS[c] (n=8)**	**Total[c] (N=44)**
**Patients with any TEAE, n (%)**	31 (81.6)	11 (100.0)	47 (87.0)	17 (51.5)	5 (62.5)	24 (54.5)
**Most common TEAEs,[d] n (%)**						
Dizziness	9 (23.7)	4 (36.4)	13 (24.1)	1 (3.0)	1 (12.5)	2 (4.5)
Fatigue	6 (15.8)	3 (27.3)	9 (16.7)	2 (6.1)	0 (0.0)	2 (4.5)

A patient with ≥2 TEAEs in the same category is only counted once

[a]The n numbers for the Titration Period are the number of patients in the SAS

[b]The n numbers for the Maintenance Period are the number of patients who took perampanel during the Maintenance Period

[c]FOS includes patients with FOS only (with or without FBTCS); GTCS includes patients with GTCS only. The "Total" column includes patients with FOS only, GTCS only, and mixed FOS and GTCS; therefore, the total number of patients is greater than the sum of patients with FOS only or GTCS only

[d]TEAEs that occurred in ≥10% of patients in the total population

FBTCS focal to bilateral tonic-clonic seizures, *FOS* focal-onset seizures, *GTCS* generalized tonic-clonic seizures, *SAS* safety analysis set, *TEAE* treatment-emergent adverse event

Supplementary Table 3. PSQI and QOLIE-31 individual domain scores at 12 months by seizure type (Safety Analysis Set)

	**FOS[a] (n=38)**	**GTCS[a] (n=11)**	**Total[a] (N=54)**
**PSQI scores, mean (SD) change from baseline at 12 months**			
Subjective sleep quality	-0.2 (0.9); n=23	0.2 (0.4); n=6	-0.1 (0.8); n=30
Sleep latency	-0.1 (1.1); n=23	-0.5 (0.6); n=6	-0.2 (1.0); n=30
Sleep duration	-0.1 (1.6); n=15	-0.3 (0.5); n=4	-0.1 (1.4); n=20
Habitual sleep efficiency	0.2 (1.4); n=15	-0.5 (1.7); n=4	0.2 (1.6); n=20
Sleep disturbances	0.1 (0.6); n=23	-0.2 (0.4); n=6	0.0 (0.6); n=30
Use of sleeping medication	-0.1 (0.8); n=23	0.0 (0.0); n=6	-0.1 (0.7); n=30
Daytime dysfunction	-0.1 (0.7); n=23	-0.2 (0.8); n=6	-0.1 (0.7); n=30
**QOLIE-31 scores, mean (SD) change from baseline at 12 months**			
Seizure worry	6.8 (25.5); n=24	7.8 (9.4); n=4	8.2 (24.2); n=29
Overall QoL	-2.4 (22.6); n=24	18.1 (18.2); n=4	0.2 (22.6); n=29
Emotional well-being	0.7 (18.2); n=24	7.0 (10.0); n=4	1.8 (17.0); n=29
Energy/fatigue	1.3 (16.8); n=24	6.3 (4.8); n=4	2.1 (15.4); n=29
Cognitive	-2.1 (13.2); n=24	-7.1 (9.2); n=4	-2.4 (12.6); n=29
Medication effects	2.8 (26.1); n=23	32.0 (25.4); n=4	4.8 (29.8); n=28
Social function	7.5 (18.8); n=24	14.3 (24.5); n=4	7.8 (19.4); n=29

Only patients with non-missing data at both baseline and the relevant post-baseline visit are included in the change from baseline summary statistics

[a]FOS includes patients with FOS only (with or without FBTCS); GTCS includes patients with GTCS only. The “Total” column includes patients with FOS only, GTCS only, and mixed FOS and GTCS; therefore, the total number of patients is greater than the sum of patients with FOS only or GTCS only

FBTCS focal to bilateral tonic-clonic seizures, *FOS* focal-onset seizures, *GTCS* generalized tonic-clonic seizures, *PSQI* Pittsburgh Sleep Quality Index, *QoL* Quality of Life, *QOLIE-31* Quality of Life in Epilepsy Inventory-31, *SD* standard deviation

Supplementary Table 4. Incidence of TEAEs and change in EpiTrack® total score in patients from ELEVATE with a history of psychiatric or behavioral events (Safety Analysis Set)

	Patients with a history of psychiatric or behavioral events			Overall population		
	**FOS[a] (n=17)**	**GTCS[a] (n=4)**	**Total[a] (N=24)**	**FOS[a] (n=38)**	**GTCS[a] (n=11)**	**Total[a] (N=54)**
**Patients with any TEAE, n (%)**	16 (94.1)	4 (100.0)	23 (95.8)	32 (84.2)	11 (100.0)	48 (88.9)
**Patients with psychiatric TEAEs, n (%)**	7 (41.2)	2 (50.0)	10 (41.7)	13 (34.2)	3 (27.3)	18 (33.3)
**Mean (SD) change in EpiTrack® total score[b] at 12 months[c]**	-1.0 (3.0); n=9	2.0 (NA); n=1	-1.1 (3.1); n=11	-0.6 (3.3); n=22	1.6 (1.7); n=5	-0.4 (3.3); n=28

A patient with ≥2 TEAEs in the same category is only counted once

[a]FOS includes patients with FOS only (with or without FBTCS); GTCS includes patients with GTCS only. The "Total" column includes patients with FOS only, GTCS only, and mixed FOS and GTCS; therefore, the total number of patients is greater than the sum of patients with FOS only or GTCS only

[b]Age-corrected total score derivation: 16–20 years = +1 point; 21–35 years = no correction; 36–45 years = +1 point; 46–50 years = +3 points; 51–65 years = +4 points; 66–70 years = +6 points; >70 years = +7 points

[c]Only patients with non-missing data at both baseline and 12 months are included in the change from baseline summary statistics

FBTCS focal to bilateral tonic-clonic seizures, *FOS* focal-onset seizures, *GTCS* generalized tonic-clonic seizures, *NA* not applicable, *SD* standard deviation, *TEAE* treatment-emergent adverse event

Supplementary Fig. 1 Median percent reduction from baseline in seizure frequency per 28 days during the (a) Titration Period (LOCF) and (b) Maintenance Period (LOCF) (Full Analysis Set)

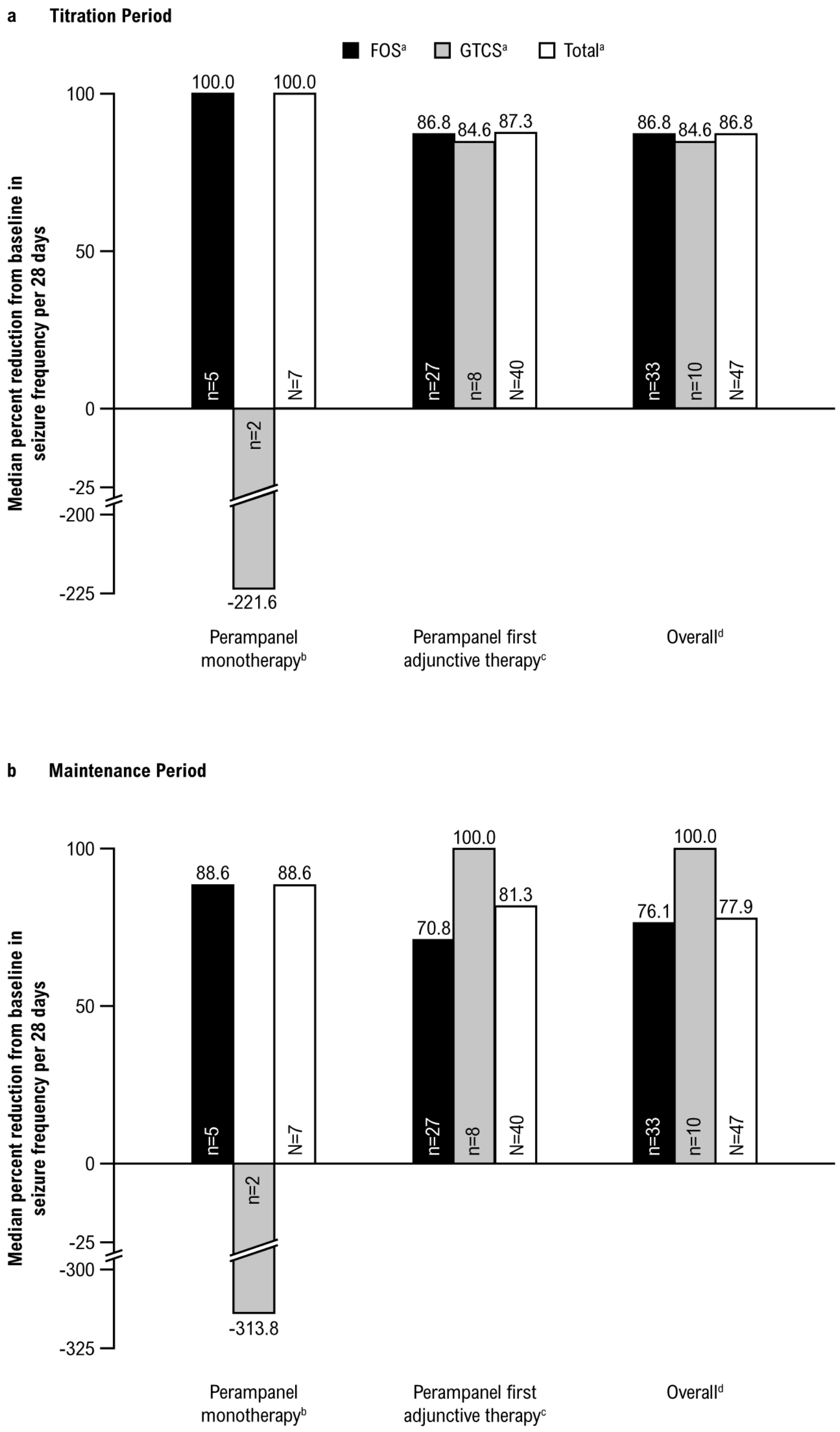

[a]FOS includes patients with FOS only (with or without FBTCS); GTCS includes patients with GTCS only. The “Total” column includes patients with FOS only, GTCS only, and mixed FOS and GTCS; therefore, the total number of patients is greater than the sum of patients with FOS only or GTCS only

[b]Patients who either received no ASMs at baseline or received another ASM and converted to perampanel only at baseline. One patient was excluded from the monotherapy subgroup analyses due to the addition of an ASM along with perampanel on the first day of treatment

[c]Patients who received perampanel as first adjunctive therapy at baseline

[d]Patients who either received perampanel monotherapy or perampanel as first adjunctive therapy at baseline

ASM anti-seizure medication, *FBTCS* focal to bilateral tonic-clonic seizures, *FOS* focal-onset seizures, *GTCS* generalized tonic-clonic seizures, *LOCF* last observation carried forward
